# Understanding the effect of wet etching on damage resistance of surface scratches

**DOI:** 10.1038/s41598-018-19716-0

**Published:** 2018-01-22

**Authors:** Benoit Da Costa Fernandes, Mathilde Pfiffer, Philippe Cormont, Marc Dussauze, Bruno Bousquet, Evelyne Fargin, Jerome Neauport

**Affiliations:** 1 CEA, DAM, CEA-CESTA, F33114 Le Barp, France; 2 Institut des Sciences Moléculaires, Université de Bordeaux, UMR 5255 CNRS, 33405 Talence, France; 3 Centre Lasers Intenses et Applications, UMR 5107 CNRS, Université de Bordeaux, CEA, 33405 Talence, France; 4 Institut de Chimie de la Matière Condensée de Bordeaux, UMR 5026 CNRS, Université de Bordeaux, 33608 Pessac, France

## Abstract

Fused silica optics often exhibit surface scratches after polishing that radically reduce their damage resistance at the wavelength of 351 nm in the nanosecond regime. Consequently, chemical treatments after polishing are often used to increase the damage threshold and ensure a safe operation of these optics in large fusion-scale laser facilities. Here, we investigate the reasons for such an improvement. We study the effect of an HF-based wet etching on scratch morphology and propose a simple analytic model to reflect scratch widening during etching. We also use a finite element model to evaluate the effect of the morphological modification induced by etching on the electric field distribution in the vicinity of the scratch. We evidence that this improvement of the scratch damage resistance is due to a reduction of the electric field enhancement. This conclusion is supported by secondary electron microscopy (SEM) imaging of damage sites initiated on scratches after chemical treatment.

## Introduction

Fused silica optics located in the final optic assembly of high-power fusion-class laser facilities, such as the National Ignition Facility^1^, SG-III^2^ or the Megajoule laser^3^, are subjected to fluence in the 5 to 14 J/cm² range at the wavelength of 351 nm, with pulse duration of a few nanoseconds. Under such irradiation conditions, fused silica optics are prone to damage. Damage sites initiate as 10 to 100 µm diameter craters on the back surface of the optical component. Damage sites grow exponentially under iterative laser shots, forcing the optics to be replaced. Previous works have shown that these damage sites are mostly triggered by residual subsurface damage or by absorbing impurities (cerium, aluminum, etc.) through the polishing process^4,5^. Polishing processes were therefore modified to reduce the existence of such precursors; damage densities of 0.01/cm² at 14 J/cm², 3 ns could then be reached^6^. At this step, if a bare polished surface is able to sustain high fluences, scratches and digs are the weak points that lead to potential damage sites. This deleterious impact of scratches on the damage threshold at 351 nm was reported by Salleo^7^. 7 µm wide scratches located on the exit surface reduced the threshold by a factor of two; larger scratches lead to a threshold as low as 5 J/cm². On the first attempts, it is possible to specify the scratch width in order to finish the part, to reduce the occurrence of large scratches. In practice, for meter-scale optics produced in large quantities, such as that used in the aforementioned laser facilities, it is difficult to ensure parts free of scratches narrower than 20 µm approximately. Acid etching can be used as a final step of the finishing process. Performed with material removal of some hundreds of nanometers to a few micrometers, this surface leaching has two different effects. It is efficient to remove the Beilby layer and thus metallic impurities embedded in this layer and beyond^8,9^, the concentration of which is higher in surface defects such as scratches^10^. It also changes the scratch morphology by opening narrow fractures, which lead to an improvement of the damage threshold^11,12^. A step forward can be made by removing a higher amount of material by wet etching (10 to 30 µm approximately). First results were obtained with a buffered oxide etch^11,13^. Similar effects were reported with HF etching^14^ and KOH etching^15^ as well. Although the positive impact of pronounced wet etching on the scratch damage threshold at 351 nm is unquestionable, the physical mechanisms explaining this effect are more elusive. The aim of this paper is to explore this question. After chemical etching of more than approximately one micrometer, the fused silica optics surface is almost free of metallic impurities (Ce, Al, Cu, Fe, etc.) induced by the polishing process^8,10^. Such precursors are therefore unlikely to be responsible for the damage initiation in etched scratches. Structural defects in damage initiation, such as NBOHC (Non Bridging Oxygen Hole center), ODC (Oxygen Deficient Center) or others^16^, were observed around surface defects (indentation, scratches). An unexplained green photoluminescence was also evidenced^17,18^ and potentially involved in the damage initiation process. However, these photo luminescent defects were removed by an HF-based etch of 1 to 2 µm^11,19^. The impact of scratch morphology on the electric field distribution and its modification after etching is therefore likely to be responsible for this increase in the threshold. This idea was first suggested by Genin^20^ with a numerical simulation carried out on 100 nm-wide and 1 µm-deep scratches. Similar results were obtained more recently on trailing indent scratches with comparable sizes^21^. In what follows, we study this relation in more detail and on a range of scratch widths representative of those found on full-scale optics; i.e., 1 to 20 µm. For this purpose, we deliberately performed scratches on fused silica optics polished surfaces. The morphology of these scratches was measured and followed during HF/HNO_3_ etching with a material removal of 12 µm. An analytical model was developed to explain the change in shape. These results were used to build a finite element model, in order to estimate the field distribution before and after etching. Laser damage tests on etched scratches were finally performed to assess the predominance of field enhancement in the damage process.

## Results and Discussions

### Scratch morphology

**Figure 1:**
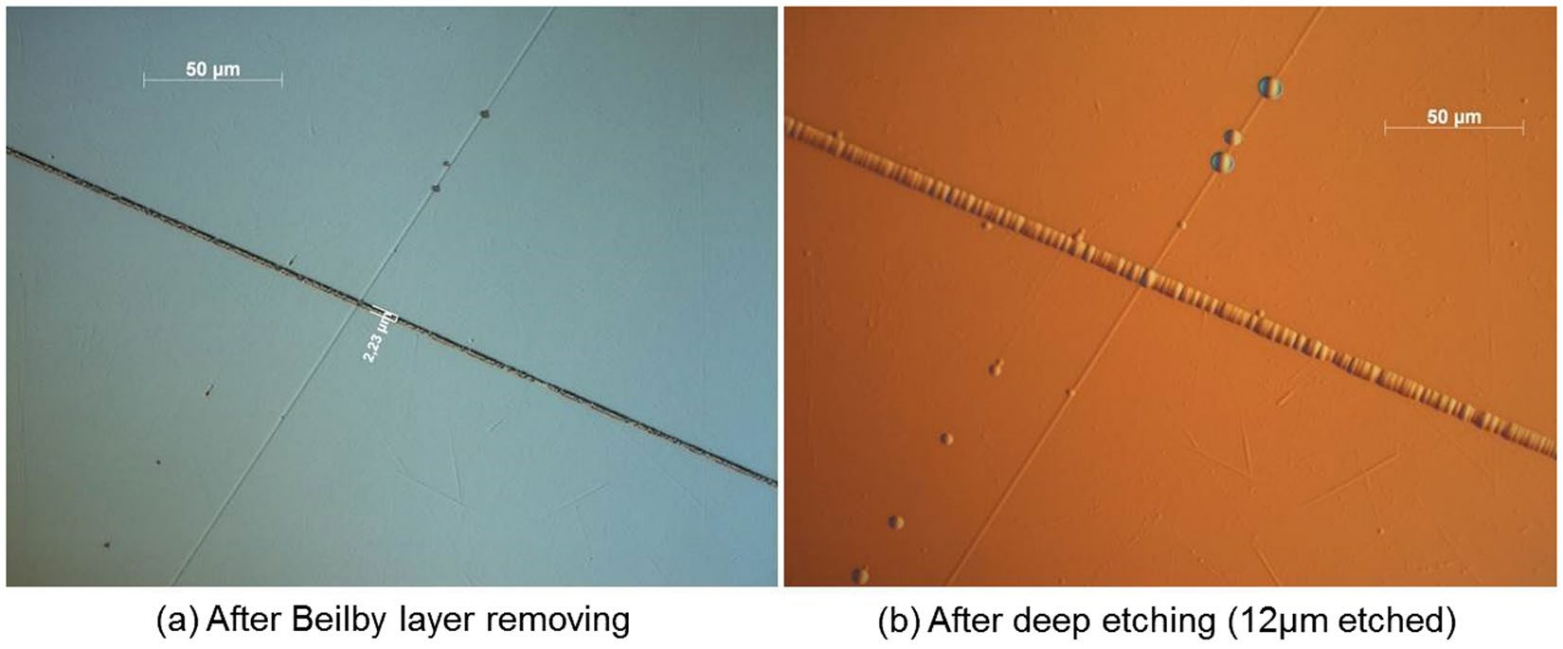
Optical microscope observations of brittle and plastic scratches (**a**) before and (**b**) after 12 µm deep acid etching.

Artificial scratches were made on high-damage-threshold fused-silica polished optical surfaces. The intentionally scratched surfaces were characterized by optical microscopy after the Beilby layer removal and after deep wet etching using an HF/HNO_3_ mixture. Deep etching material removal was 12 µm. Figure 1 presents the typical morphology modification observed in scratches. Both brittle and plastic scratches are observed.

**Figure 2:**
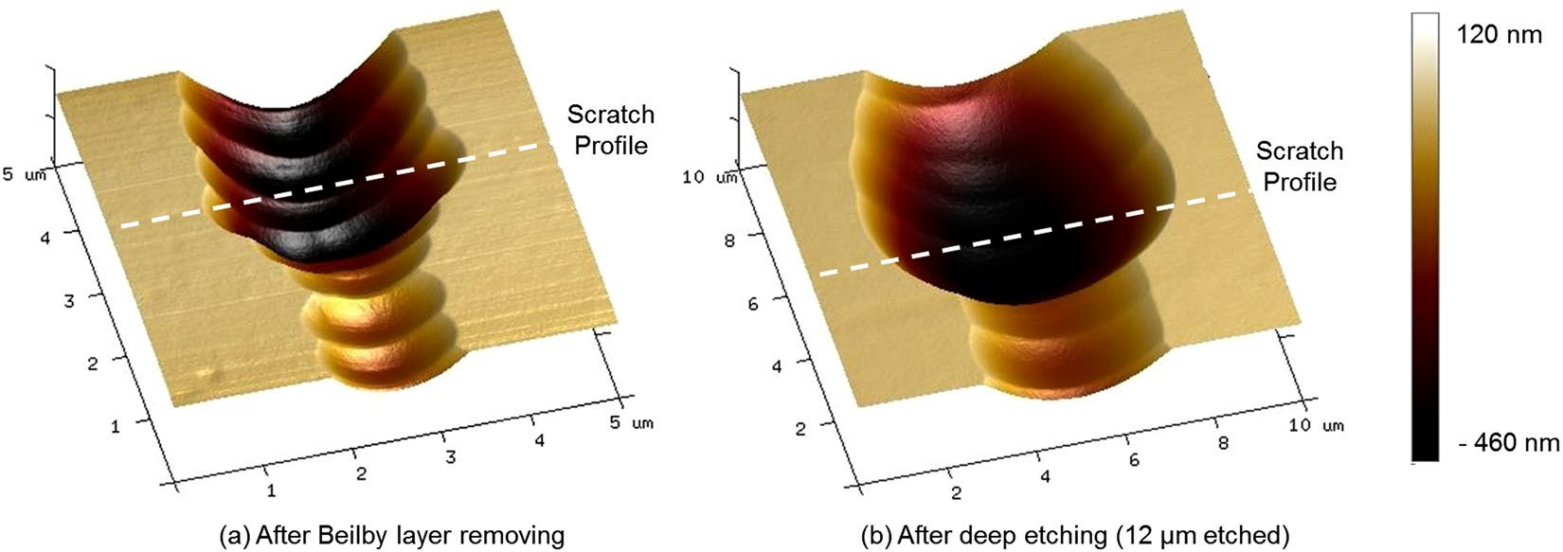
AFM observations of a brittle scratch (**a**) before and (**b**) after 12 µm deep acid etching. Dotted lines correspond to the profiles presented in Fig. 3.

Microscopic observations (Fig. 1) reveal that the morphology of plastic scratches does not change during the 12 µm deep etching, whereas brittle scratches get wider. AFM observations were performed on brittle scratches to evaluate the widening and to analyze this morphology change in more detail. A three-dimensional representation obtained from an AFM characterization of one of the brittle scratches is presented in Fig. 2.

**Figure 3:**
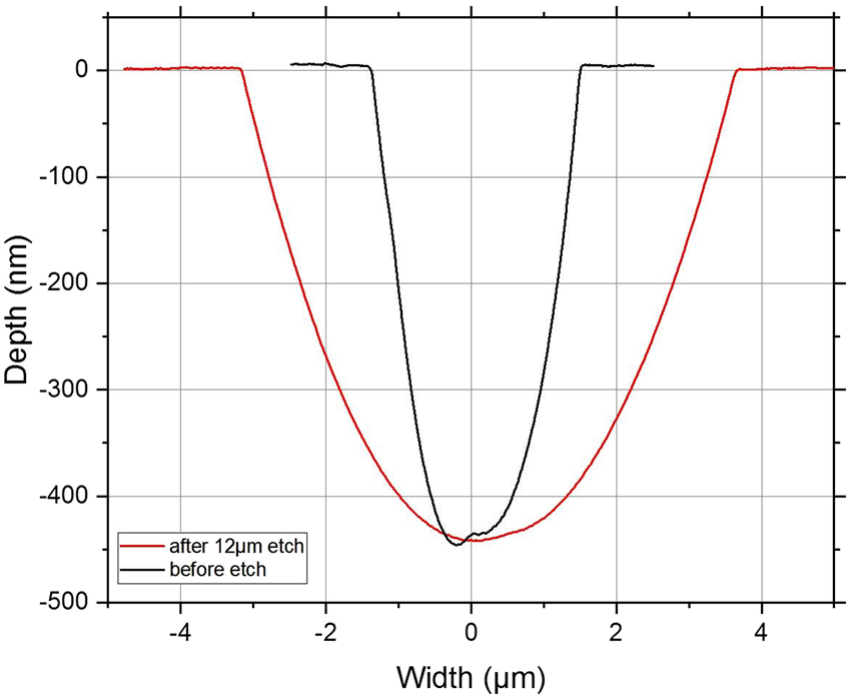
Scratch depth profiles before and after 12 µm deep acid etching, obtained by post treatment from the AFM images exposed in Fig. 2.

The dotted lines of Fig. 2 correspond to the scratch sections displayed in Fig. 3. The AFM characterization in Fig. 2(a) shows an irregular morphology of the brittle scratch after the initial shallow wet etch. The scratch bottom is particularly bumpy with spatial frequency features. After 12 µm deep etching, the brittle scratch has broadened by some micrometers and its morphology appears more regular. Scratch bottom and edges are smoothed. Depth profiles displayed on the Fig. 3 demonstrate that the depth is kept unchanged after the deep wet etching.

**Figure 4:**
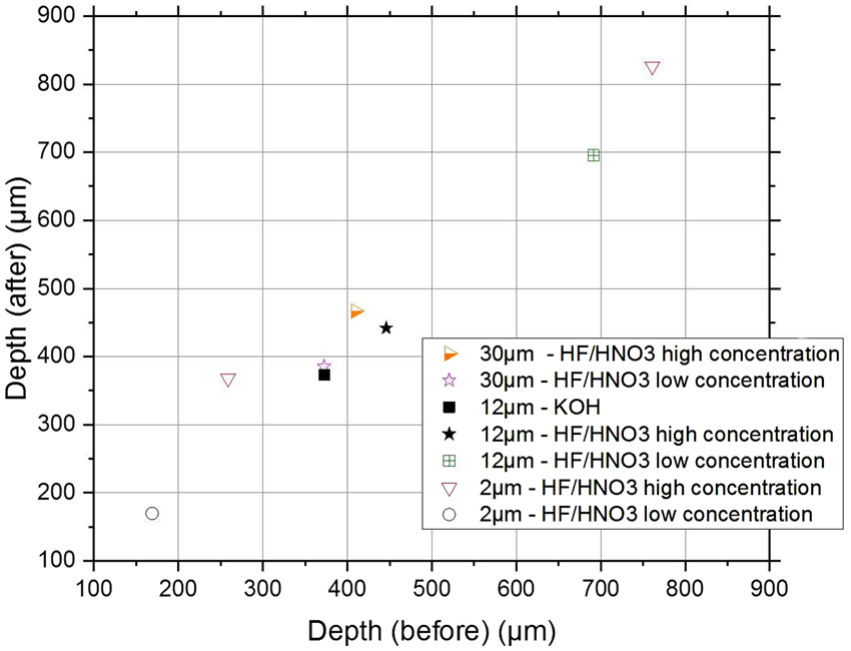
Scratch depth before and after etching. Wet etching is conducted with different etching solutions using various concentrations of HF/HNO_3_ and KOH and different material removals.

Several scratched samples were etched under different conditions (depth removed, HF/HNO_3_ or KOH chemical solutions and etching rate). Scratch morphologies were characterized using AFM as described above. AFM measurements confirmed that scratch depths remain unchanged before and after deep etching (See Fig. 4).

We also evidenced that scratch widening is proportional to the material removal only. Neither the chemical solution used to perform etching nor the etching rate has influence on the morphology change. This is the topic of the next section.

### A kinematic model of surface evolution during chemical etching

**Figure 5:**
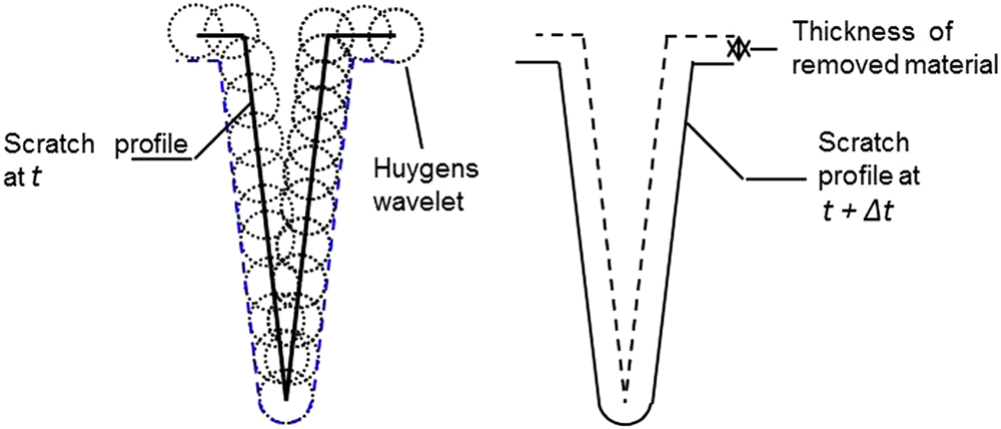
Modeling of chemical erosion using Huygens wavelets for a simplified scratch.

In terms of kinematic evolution, chemical etching is comparable to surface erosion. As was described by Katardjiev^22^, the erosion process of a smooth surface can be described by the Huygens principle of wave-front propagation; i.e., every point on the surface acts as an elementary source of secondary disturbances (Huygens wavelets). The form of the wavelet depends on the anisotropy ratio of the chemical reaction. Due to its amorphous nature, fused silica is removed by chemical reaction with no specific spatial direction^23^. Thus, we consider that the silica removal is isotropic and also does not depend on time. The erosion process described with Huygens wavelets is illustrated in Fig. 5 for a simplified scratch.

**Figure 6:**
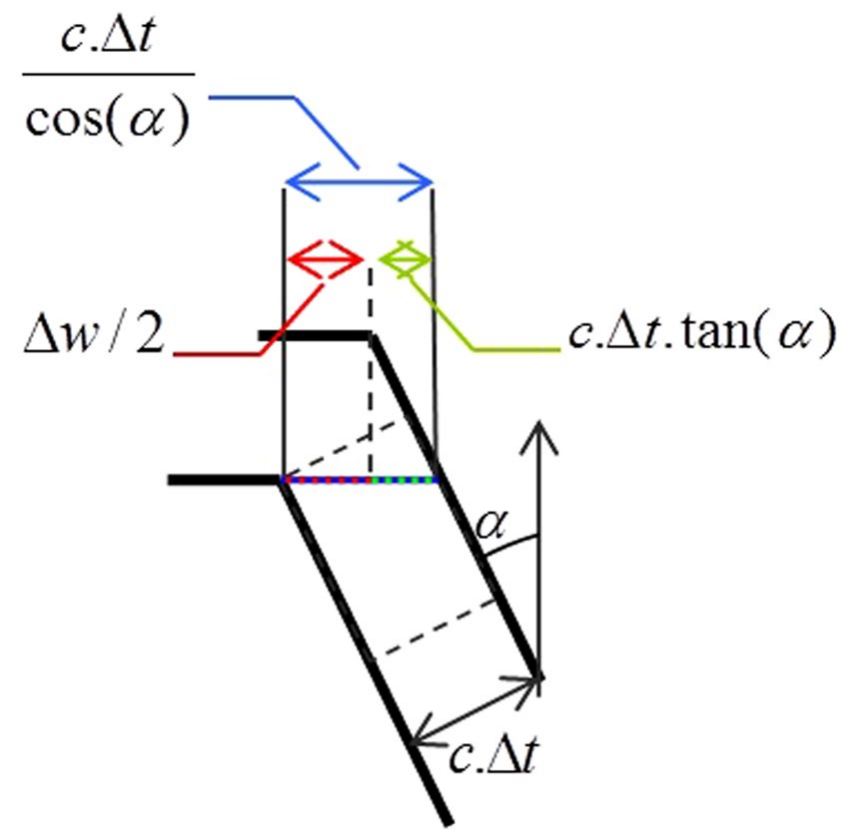
Geometrical approach of the widening of the scratch.

Thanks to this model, the surface evolution, which is a relation between the dimensions of the scratch (slope, depth, width) and the etching rate, can be estimated by geometrical calculation. As can be seen in Fig. 6, the widening (crack opening) of the scratch can be calculated by the following relation:

1 $${\rm{\Delta }}{\rm{w}}=2.\,{\rm{c}}.\,{\rm{\Delta }}{\rm{t}}.(\frac{1}{\cos ({\rm{\alpha }})}-\,\tan ({\rm{\alpha }}))$$

Where *c* is the etching rate, *Δt* is the etching time and *α* is the slope of the scratch profile, which can be expressed by:

2 $$\cos ({\rm{\alpha }})=\frac{{\rm{d}}}{\sqrt{{{\rm{d}}}^{2}+{(\frac{{\rm{w}}}{2})}^{2}}}$$

Where d is the depth of the scratch and w is its width before etching.

If we consider the ratio R between the width and the depth (R = w/d), we obtain a simple relation giving the widening of the scratch in function of the etching rate c, the etching time Δt and the ratio R:

3 $${\rm{\Delta }}{\rm{w}}=2.\,{\rm{c}}.\,{\rm{\Delta }}{\rm{t}}.(\sqrt{{(\frac{{\rm{R}}}{2})}^{2}+1}-\frac{{\rm{R}}}{2})$$

**Figure 7:**
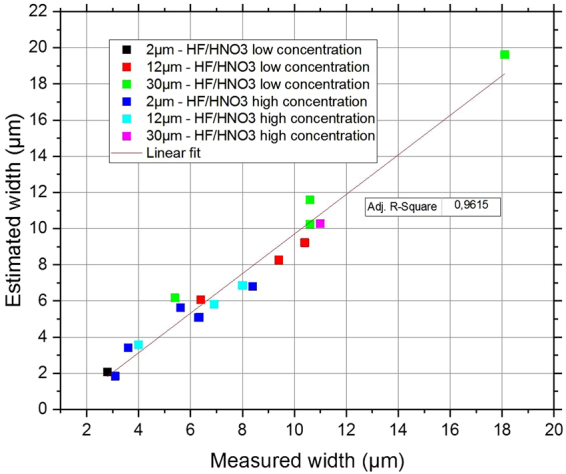
Width of various scratches after etching with different parameters. Comparison between measurements and predictions using the analytic relation of Equation (3). A linear fit is evidenced with R² = 0.9615.

Equation (3) was used to predict the scratch widening in a variety of scratches after HF/HNO_3_ wet etching, using either a low-concentration solution or a high-concentration solution, and different removal depth (2 µm, 12 µm and 30 µm). The results are summarized in Fig. 7. We demonstrate a perfect prediction of the scratch widening whatever the etching parameters.

In the typical case of a plastic scratch, its depth is very small, which results in a very high ratio R. For high values of ratio R, the widening of the scratch tends to zero as can be seen in Equation (3). Thus, this explains why the morphology of plastic scratches does not change during etching, as was noticed in Fig. 1. Another typical case is a deep scratch. For such a scratch, the ratio R tends to zero and, according to Equation (3), the widening equals twice the amount of the base thickness removed. After etching, the ratio R increases because the width increases, and the depth does not change. Thus, for long-time etching, the ratio R increases as a function of time and the widening decreases and tends to zero, as expressed by Equation (3). Thus, finally this can explain the behavior described by Wong *et al*.^24^, where the half-width of the crack was found to etch slower than the base surface removal.

### Light intensification by scratches

**Figure 8:**
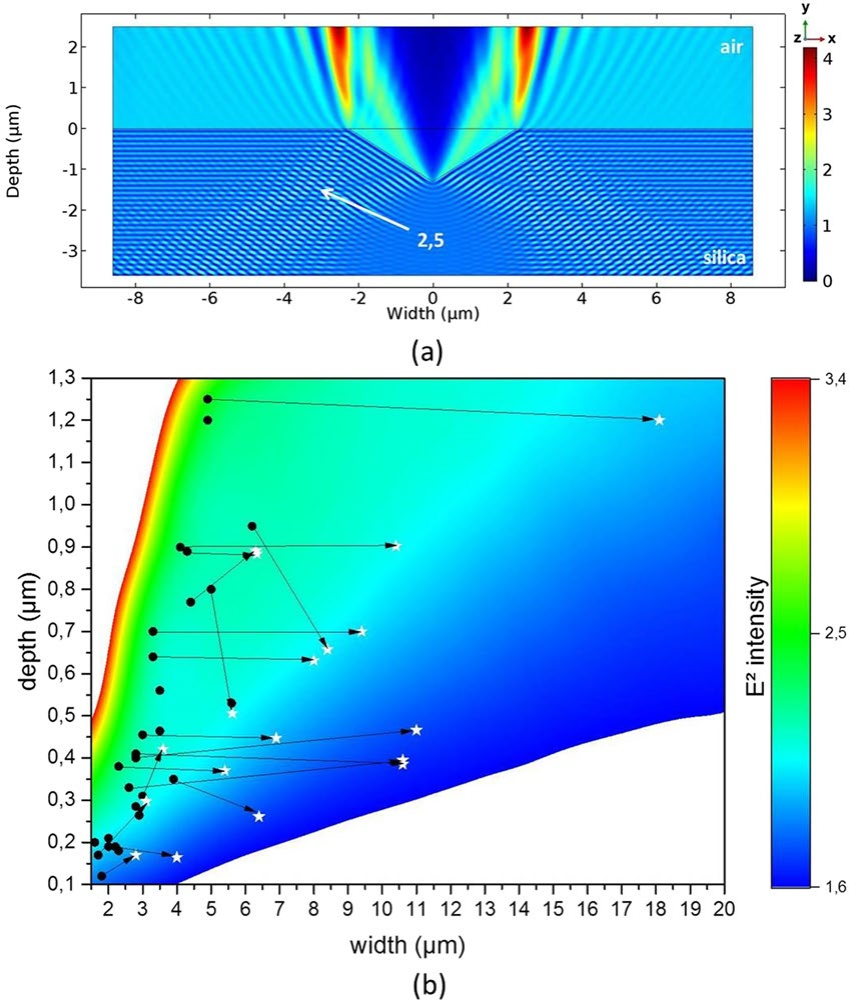
(**a**) Typical intensity (E²) distribution around a triangle-shaped scratch in TE polarization; (**b**) Evolution of the intensity maximum in the fused silica induced by a triangle-shaped scratch as a function of triangle depth and width. Intensity maximum was obtained by simulations performed under TE polarization. Circles correspond to the depth/width of real scratches before etching. Stars represent the scratch width/depth after chemical etching with different etching conditions (detailed in Figs 6 and 7).

Scratches on the surface of an optical component disrupt the propagation of the laser beam by causing interferences. These interferences can create a local increase in the electric field intensity, which can trigger laser damage. To explain the origin and location of laser damage and the improvement of the laser-induced damage threshold after a chemical etching, we carried out numerical simulations with a finite-element method. A first approach was conducted by modeling the scratch with a triangle shape. This choice was motivated by the fact that the angle formed between the surface and a real scratch increases with the widening induced by chemical etching. 2D simulations were performed to compute the electric field distribution while varying width and depth of the triangle-shaped scratch in a range similar to that found on real optical components. A typical intensity distribution around a triangle-shaped scratch is presented in Fig. 8(a).

Figure 8(b) shows the evolution of the maximum intensity E², as a function of the width and depth of the triangle-shaped scratch, in TE polarization. We observe that when the width-to-depth ratio decreases, the amplitude of the intensity maximum in silica increases. Indeed, the reflection coefficient depends on the angle of incidence between the incident beam and the silica-air interface. Like this angle, the intensity of the reflected wave increases and consequently the maximum of the intensity increases^20^. For the TM polarization, we observe the same evolution of the intensity maximum as a function of the reflection coefficient at the silica-air interface, as already reported elsewhere for high-aspect-ratio triangle-shaped scratches^20^. We have also plotted in the same figure the experimental values of the width and depth before (circles) and after (stars) wet etching of the real scratches. These scratches are those already presented in Figs 6 and 7. We observe that if we approximate real scratches as triangles, chemically untreated scratches, described by the points, have an intensity maximum ranging from 1.9 to 2.5. Wet chemical etching, described by the stars, increases the scratch width-to-depth ratio, and leads to a decrease in the intensity enhancement in fused silica. However, this reduction of intensity enhancement is limited compared to the laser damage improvement previously reported^11,13,15,25^. Indeed, modeling a scratch by a triangle is too great an approximation. For example, local features existing in the scratch that could change the field distribution and are shown in Fig. 2 are not taken into account. We have thus carried out finite-element numerical calculations on real scratch profiles, obtained by AFM mapping, to account for the influence of these irregularities on the intensity of the electric field in the vicinity of the scratch.

**Figure 9:**
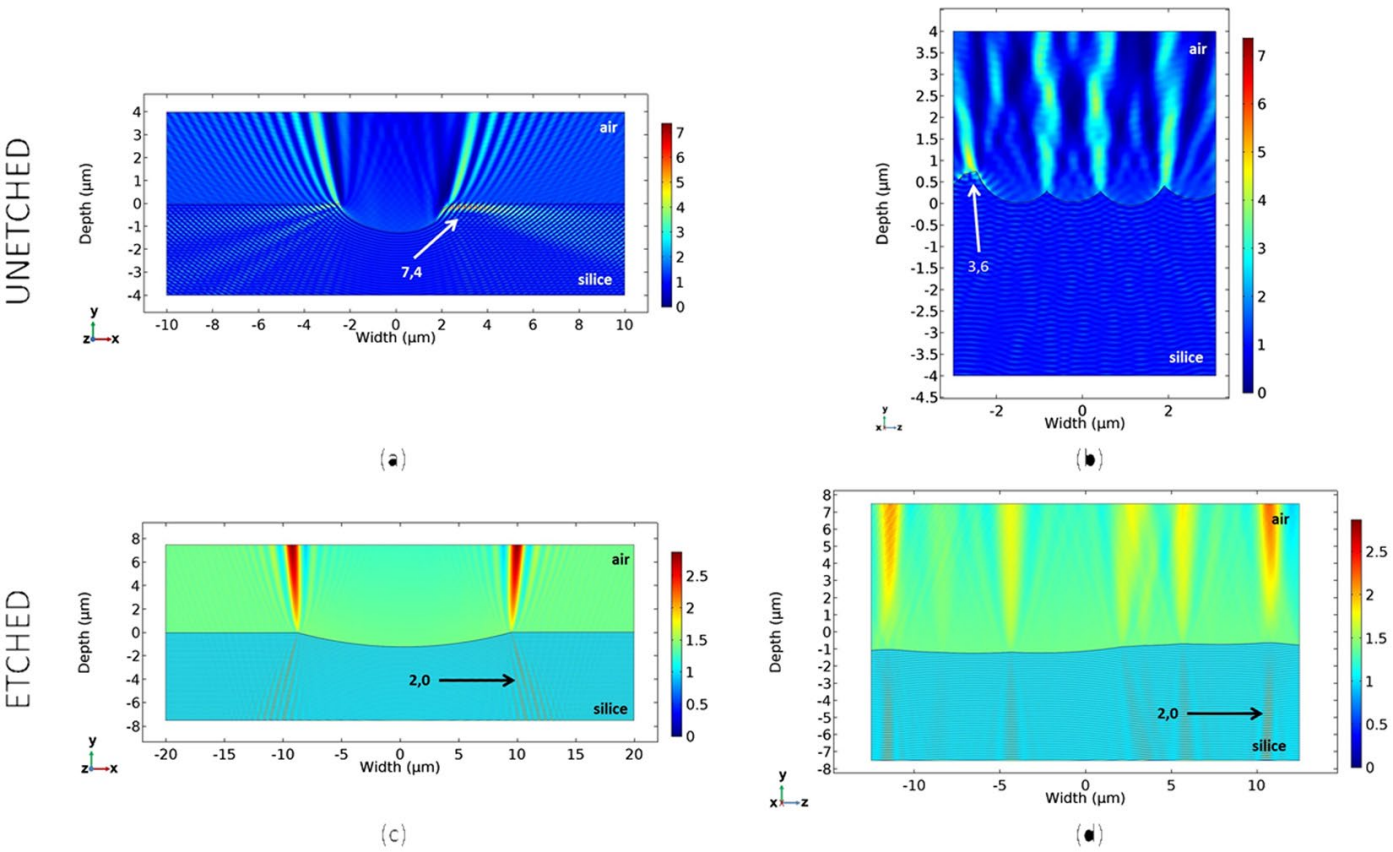
Evolution of the intensity (E²) distribution on an AFM cross-section perpendicular ((**a**) and (**c**)) or centered parallel ((**b**) and (**d**)), before ((**a**) and (**b**)) and after ((**c**) and (**d**)) a 12 µm HF/HNO_3_ wet etch. 2D finite-element modeling in TE polarization. Maximal intensity is decreased by the deep wet etching.

Figure 9 shows, in TE polarization, the electrical field intensity mapping of two cross-sections (perpendicular and parallel centered respectively) from the same scratch, before and after 12 µm deep chemical HF/HNO_3_ etching. On the chemically untreated scratch (Fig. 9a and b), we observe the existence of multiple points whose intensity varies greatly according to the local irregularities of the scratch. Moreover, the intensity maximum is greatly enhanced compared to the triangular shape approximation. On chemically etched scratches (Fig. 9c and d), we observe the presence of intensity maxima of lower amplitude than those previously seen. Indeed, the suppression of irregularities on the walls and bottom of the scratch and the reduction of the incidence angle between the incident beam and the silica-air interface reduces the reflection coefficient and consequently decreases the intensity of the reflected wave. We also performed this analysis in TM polarization; although the amplitude of the intensity maximum for chemically untreated scratches is lower than that calculated in TE polarization, we observe a decrease in the same trend; i.e., a reduction of the intensity maximum during the deep chemical etching of these scratches. As a conclusion, 2D finite-element simulation on real scratch profiles show a reduction of electric field enhancement consistent with the damage improvement previously reported.

To confirm this detrimental role of field enhancement on laser damage, we performed SEM imaging of damage initiated by scratches.

**Figure 10:**
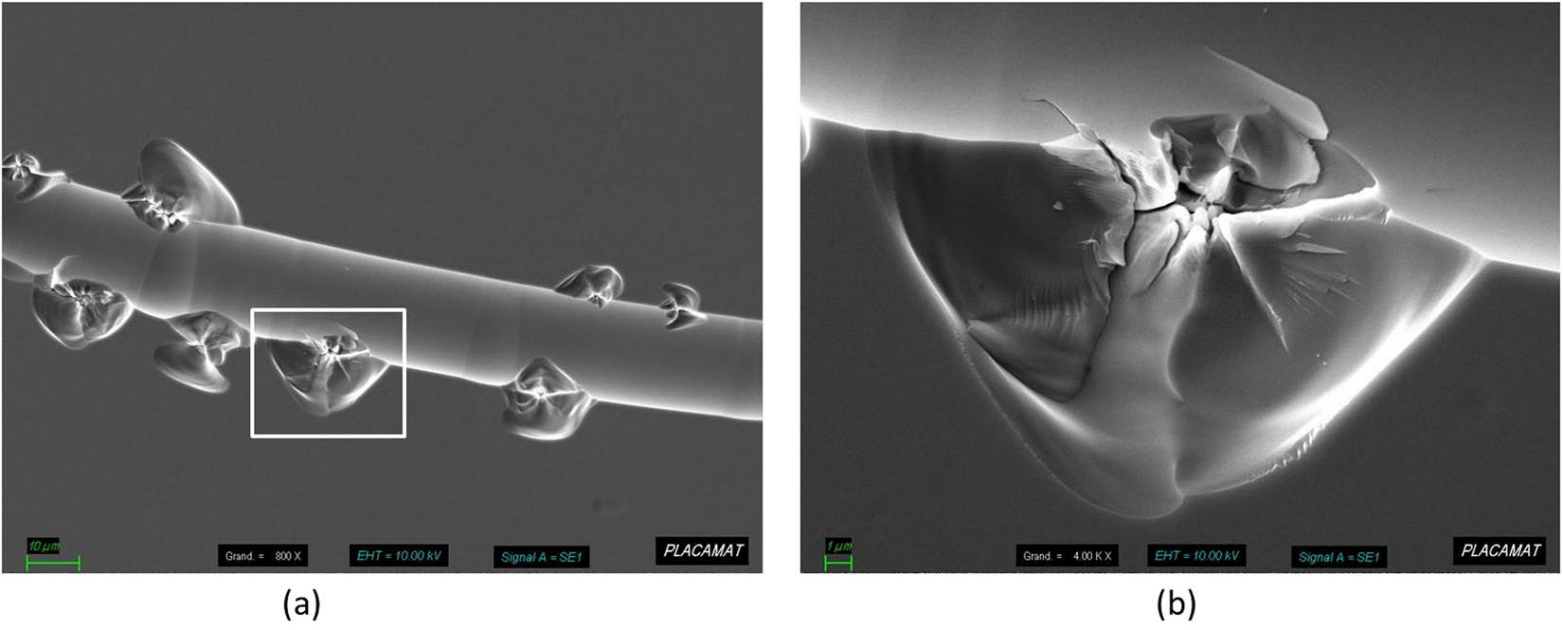
Laser damage initiation on a 12 µm wet etched scratch at 18.7 J/cm², 355 nm, 3 ns. Multiple damage sites are triggered at the edge of the scratch, were field enhancement is maximum (**a**). Close-up view of the area in the square, showing the melted region suggesting field initiation from within the fused silica.

Figure 10 presents an image obtained by SEM on a 12 µm deep chemically etched scratch after a laser damage test. Laser damage was performed with an Nd: YAG tripled 355 nm laser with a pulse duration of 3 ns. This damage was obtained at a fluence of 18.7 J/cm². Figure 10 shows that smoothing of the scratch bottom prevents damage in this area. Damage is created near the scratches in good consistence with numerical calculations carried out on these cross-sections (Fig. 9). One can notice that despite the smooth and apparently regular shape of the etched scratch observed in the SEM image, damage initiation is not uniform at the etched scratch edges as suggested by the 2D finite-element model simulation. We believe that these localized damage sites might be due to 3D interference effects that are not taken into account in the 2D modeling that we performed.

## Conclusions

In summary, we have investigated the effect of deep etching on the morphology of scratches performed on fused-silica polished surfaces. We evidenced that scratch widening follows an analytic model that is proportional to the width-to-depth ratio of the initial scratch. This model explains why shallow ductile scratches remain almost unchanged during deep etching, while brittle larger scratches are deeply modified. 2D finite-element simulation was performed to evaluate the electric field intensity distribution in the vicinity of a scratch, before and after wet chemical etching. We demonstrated that an intensity reduction of a factor close to 5 is obtained in the intensity maximum. Deep etching smooths the scratch profile and therefore significantly reduces the intensity maximum, which explains the improvement. This mechanism is supported by SEM observation, which confirms the correspondence between the intensity maxima and damage initiation.

## Materials and Methods

### Sample polishing, scratch protocol

Fused silica samples (Corning 7980, 50 mm in diameter, 5 mm thick) were manufactured with an optimized grinding process that reduces the subsurface damage^26^, followed by a pre-polishing and a super-polishing^27^. Therefore, samples exhibit a high damage threshold at 351 nm and no surface scratch remained on their surface. Scratches were then deliberately created on the sample surfaces. The scratch process consists in a 30-minute long polishing cycle using a single-side polishing machine (Logitech PM5) with polishing slurry composed of colloidal silica contaminated with cerium oxide particles. This protocol ensures the creation of 1 to 10 µm width scratches equivalent to those created during polishing on full-scale optics. Since their density on the sample surface is significantly higher than on full-scale optics, these scratches can be easily used as test-beds for wet etching experiments. After the scratch protocol, shallow wet etching (2 µm removal) was performed on the scratched side with a mixture of hydrofluoric acid HF (2.7 wt%) and nitric acid HNO_3_ (22.8 wt%) at room temperature, using the system presented in Reference^5^. This shallow etching is used to remove the polishing layer (Beilby layer) and reveal scratches masked by the Beilby layer. Samples were then cleaned in an automatic washing machine. The cycle started with a wash in an ultrasonic bath at 50 °C, using a detergent in DI water. Rinsing was performed in another ultrasonic bath in DI water at 25 °C. The process ended with DI water rinsing by overflowing. Drying was achieved by lifting out.

### Wet etching protocols

Samples were deep-etched under different conditions (acid or basic chemical solution, different etching rates and different thicknesses removed). Acid etching was performed with a mixture of hydrofluoric acid HF (6.7 wt%) and nitric acid HNO_3_ (56.6 wt%) at room temperature. Basic etching was performed in a beaker containing a KOH solution (30 wt%) heated to 100 °C. A complete description of these etching protocols is available elsewhere^15^. After acid or basic etching, samples were cleaned in the automatic washing machine, using the same protocol as that previously described.

### Scratch characterization

Scratch morphology after Beilby layer removal and after deep etching was analyzed using an optical microscope and an atomic force microscope (AFM). Optical microscope images were performed with an Axio Imager 2 (Zeiss, Jena, Germany), using the Nomarski Interference Contrast (NIC) mode and 50× magnification. AFM was a Dimension Icon (Bruker, Billerica, MA, USA) used with scan-assist air tips and Peak Force Tapping mode. The analysis areas measured 5 µm × 5 µm or 10 µm × 10 µm. Images were obtained with 512 lines and 512 samples/line, which correspond to spatial resolutions of 10 nm and 20 nm, respectively, for the 5 µm and 10 µm wide images. The scan rate was 0.2 Hz and the peak force set point was 250 pN.

### Finite-element simulations

Numerical simulations were carried out by finite-element simulation using the COMSOL software Version 5.2a and the RF module. We simulated the propagation of a planar wave from silica to the air, either through a scratch approximated by a triangle or through cross-sections of several scratches obtained by AFM imaging. The incoming wave is normal to the exit surface and its wavelength is 351 nm. TM polarization is defined as the polarization perpendicular to the scratch direction. TE polarization is the polarization parallel to the scratch direction. The index of silica is fixed at 1.45 and that of air at 1. Modeling is performed in 2D (3D modeling over a large enough domain was impossible, due to high calculation costs). We carried out the calculations of the electric field propagation in the total field, where we have imposed a diffusion condition at the boundaries of the model. To avoid parasitic oscillations at the boundaries of the model, we placed an absorbent layer around it called a PML (perfect matched layer). In order to obtain a good resolution of the mapping of the electric field standard, which is squared to obtain the intensity, the model is meshed with rectangular sub-elements of maximum dimensions λ/7.
