# Author Correction: Understanding the effect of wet etching on damage resistance of surface scratches

**DOI:** 10.1038/s41598-018-24173-w

**Published:** 2018-04-12

**Authors:** Benoit Da Costa Fernandes, Mathilde Pfiffer, Philippe Cormont, Marc Dussauze, Bruno Bousquet, Evelyne Fargin, Jerome Neauport

**Affiliations:** 1 CEA, DAM, CEA-CESTA, F33114 Le Barp, France; 2 Institut des Sciences Moléculaires, Université de Bordeaux, UMR 5255 CNRS, 33405 Talence, France; 3 Centre Lasers Intenses et Applications, UMR 5107 CNRS, Université de Bordeaux, CEA, 33405 Talence, France; 4 Institut de Chimie de la Matière Condensée de Bordeaux, UMR 5026 CNRS, Université de Bordeaux, 33608 Pessac, France

Correction to: *Scientific Reports* 10.1038/s41598-018-19716-0, published online 22 January 2018.

Marc Dussauze, Bruno Bousquet and Evelyne Fargin were omitted from the author list in the original version of this Article. This has been corrected in the PDF and HTML versions of the Article.

